# Correction: Antibody-mediated spike activation promotes cell-cell transmission of SARS-CoV-2

**DOI:** 10.1371/journal.ppat.1013394

**Published:** 2025-08-04

**Authors:** The PLOS Pathogens Staff

The following information is missing from the Funding statement: Natural Science Foundation of China (92269202, GM; 81830049, GM; 81761128012, GM).

The publisher apologizes for the error.
