# Supplementary figures and images for: Antibody-mediated spike activation promotes cell-cell transmission of SARS-CoV-2
Source: PLoS Pathog. 2023 Nov 10;19(11):e1011789. doi: 10.1371/journal.ppat.1011789 (PMC10664894; doi:10.1371/journal.ppat.1011789)

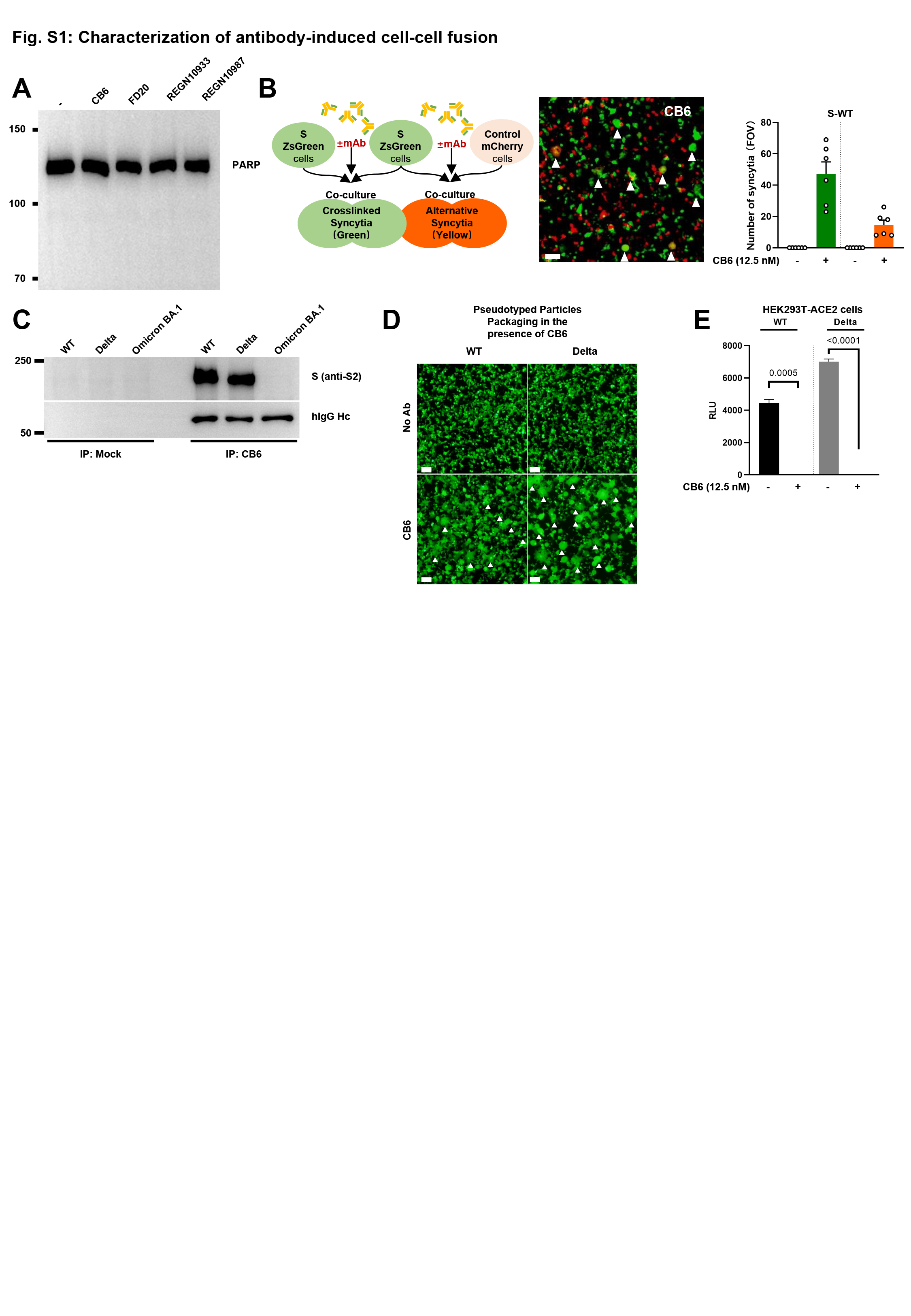

Supplement: S1 Fig — (A) Immunoblot of full-length PARP, collected from HEK293T cell expressing WT spike, stimulated by 12.5 nM CB6, FD20, REGN10933 and REGN10987 for 16 hours. Blot is representative of two individual experiments; (B) Schematics of antibody-induced cell-cell fusion among cells co-expressing spike and ZsGreen (Crosslinking syncytia, Green), or spike-to-adjacent cells expressing mCherry (Alternative syncytia, Yellow); representative images and quantification of crosslinked and alternative syncytia per field of view (FOV), stimulated without or with 12.5 nM CB6 for 16 hours, scale bars are representative of 50 μm. Data are representative of six individual repeats; (C) Immunoblots showing CB6 immunoprecipitants and input control of WT, Delta and Omicron BA.1 spike VOCs expressed in HEK293T cells. Blots are representative of two individual experiments; (D) Representative fluorescent images captured at 488 nm from HEK293T cells co-expressing MLV-gag, MLV-luc/ZsGreen, WT or Delta Spike VOCs, stimulated without or with 12.5 nM CB6 for 24 hours, scale bars are representative of 50 μm. Images are representative of two independent experiments, syncytia are indicated with white arrows; (E) Luciferase activity collected from HEK293T-ACE2 cells, after transduction by MLV-S PPs containing 12.5 nM CB6 prepared from (D). Data are representative of four individual repeats and displayed as individual points with mean ± standard error of the mean (SEM). P value was obtained by one-way ANOVA with Sidak’s post hoc test and is indicated on the figure. (TIF) [file ppat.1011789.s001.tif]

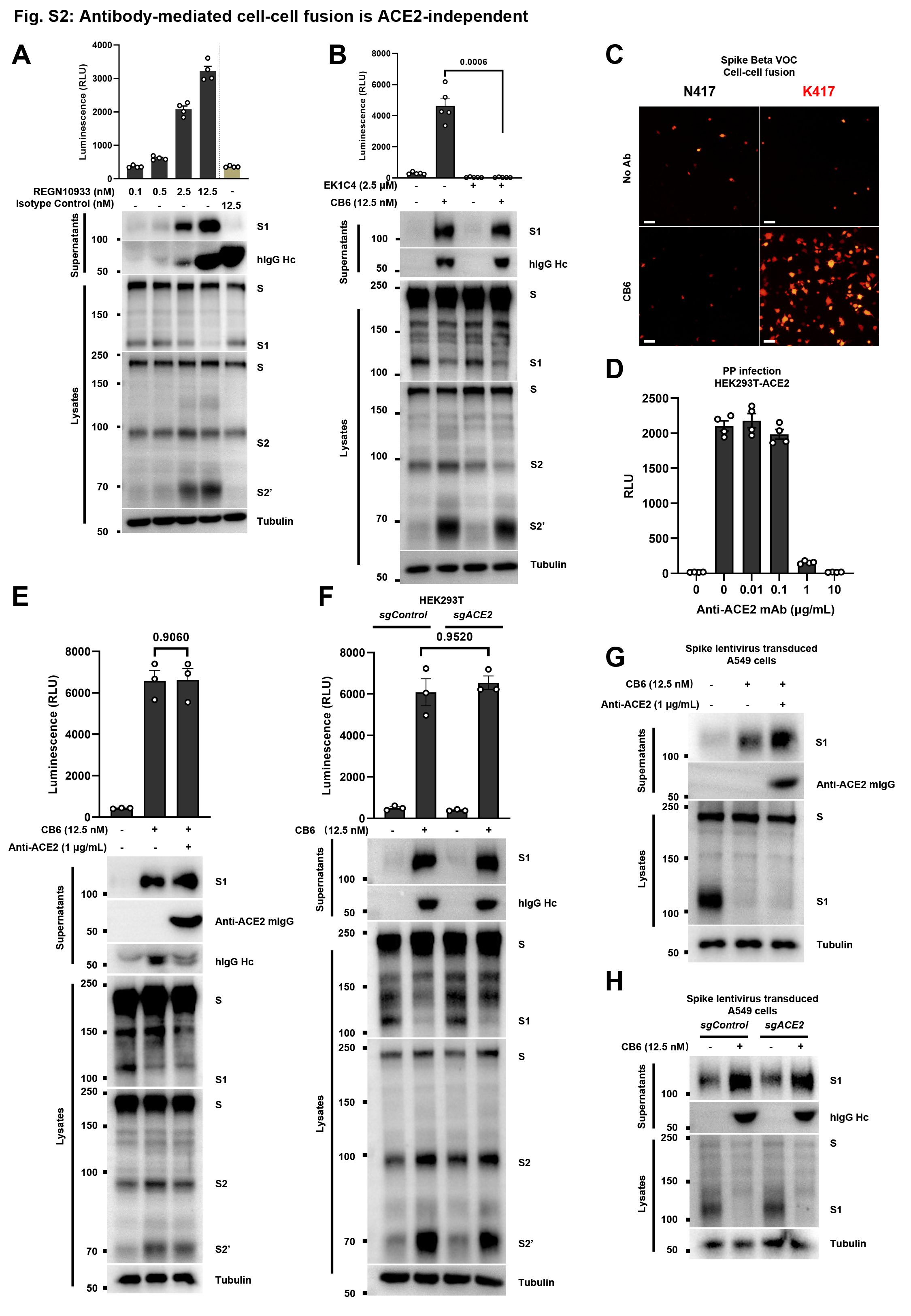

Supplement: S2 Fig — (A) Luciferase activity (RLU) measured from cell-cell fusion assay and immunoblots showing shedded S1 subunits, hIgG Hc, full-length spike, S1, S2 and cleaved S2’ collected from supernatant and cell lysate fractions of HEK293T cells stimulated with 0.1, 0.5, 2.5, 12.5 nM REGN10933 or Isotype Control for 16 hours. Data and blots were representative of four individual repeats; (B) Luciferase activity (RLU) measured from cell-cell fusion assay and immunoblots showing shedded S1 subunits, hIgG Hc, full-length spike, S1, S2 and cleaved S2’ collected from supernatant and cell lysate fractions collected of CB6-stimulated HEK293T cells, treated without or with 2.5 μM EK1C4 for 16 hours. Data and blots are representative of five individual repeats; (C) Fluorescent images of mCherry reporter cell-cell fusion assay, captured at 594 nM from HEK293T cells co-expressing Beta N417 and K417 (reversion) with Cre, mixed with stop-mCherry HEK293T cells and stimulated without or with 12.5 nM CB6 for 16 hours, scale bars are representative of 50 μm; (D) Infectivity in RLU obtained from HEK293T-ACE2 cell lysates infected with SARS-CoV-2 MLV-S-WT pseudotyped particles in the presence of 0.01, 0.1, 1 and 10 μg/mL mouse anti-ACE2 blocking antibody. Data are representative of four repeats; (E) Luciferase activity (RLU) measured from cell-cell fusion assay and immunoblots showing shedded S1 subunits, anti-ACE2 mIgG, hIgG Hc, full-length spike, S1, S2 and cleaved S2’ collected from supernatant and cell lysate fractions of HEK293T cells stimulated with 12.5 nM CB6 antibody, without or with 1 μg/mL anti-ACE2 blocking antibody; (F) Luciferase activity (RLU) measured from cell-cell fusion assay and immunoblots showing shedded S1 subunits, hIgG Hc, full-length spike, S1, S2 and cleaved S2’ collected from supernatant and cell lysate fractions of sgControl or sgACE2 HEK293T cells stimulated with 12.5 nM CB6 antibody for 16 hours. Data are representative of three individual repeats; (G) Immunoblots sh [file ppat.1011789.s002.tif]

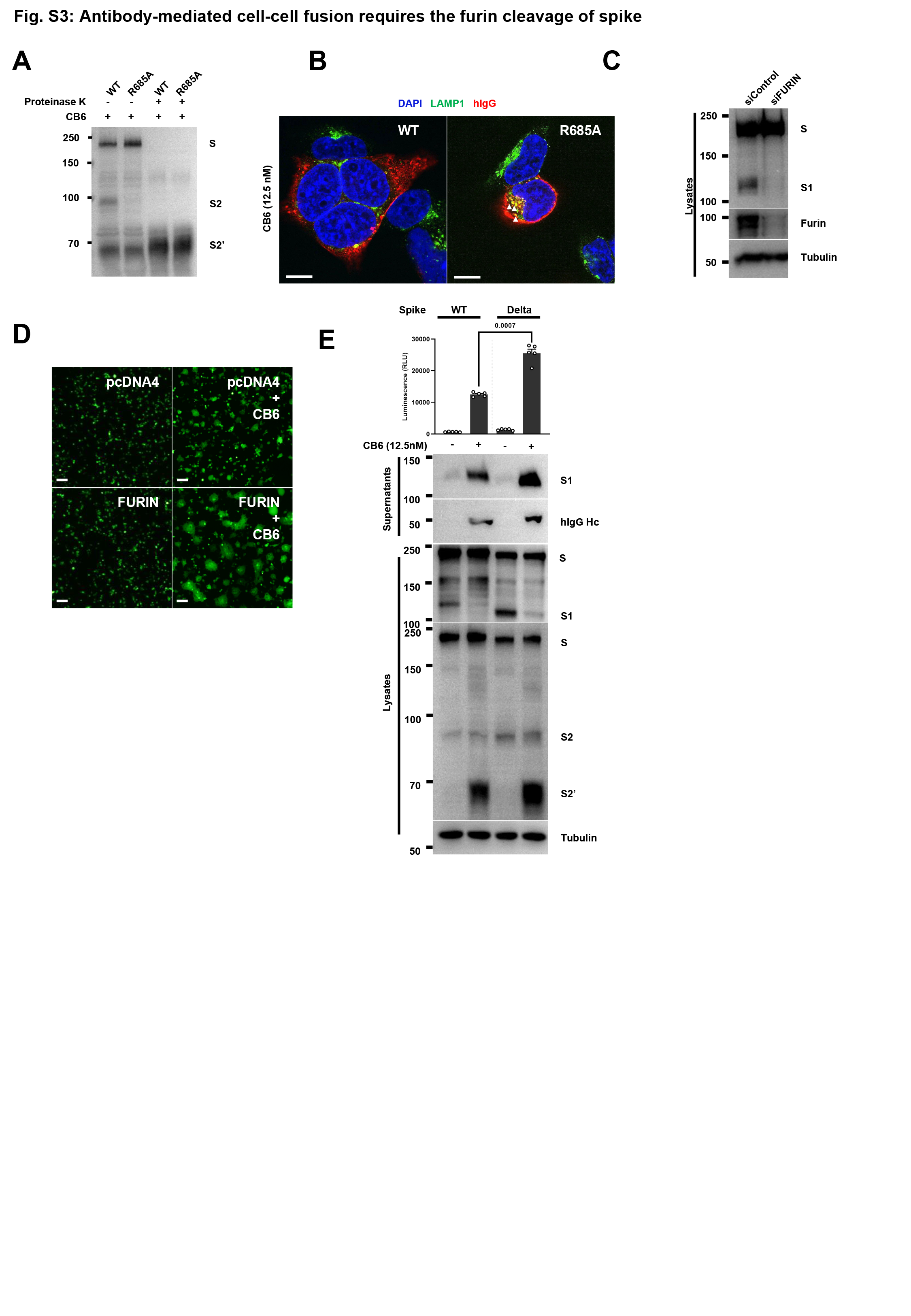

Supplement: S3 Fig — (A) Immunoblots showing proteinase K-resistant S2’ cleavage product, obtained from WT and R685A spike HEK293T cell lysates, stimulated with 12.5 nM CB6 for 16 hours. Lysates were then treated without or with 10 μg/mL proteinase K for 30 min at 37°C. Blots are representative of two individual repeats; (B) Representative confocal images of 12.5 nM CB6 antibody-stimulated HEK293T cells expressing WT or R685A spike mutant for 16 hours. Anti-LAMP1 and Anti-human IgG (H+L chains) were stained with Alexa fluor 488 and 555 respectively, co-localizations are indicated with white arrows, scale bars are representative of 10 μm. Images are representative of two individual experiments; (C) Immunoblots showing full-length S, cleaved S1, furin and tubulin, collected from HEK293T cells co-expressing 50 nM non-targeting (siControl) or human furin-targeting (siFURIN) siRNAs with full-length spike for 24 hours. Blots are representative of three individual repeats; (D) Representative fluorescent images of HEK293T cells co-expressing WT or Delta spike VOCs, co-cultured with pcDNA4 control or FURIN over-expressing cells stimulated without or with 12.5 nM CB6 antibody for 16 hours, scale bars are representative of 50 μm. Images are representative of two individual experiments; (E) Luciferase activity (RLU) measured from cell-cell fusion assay and immunoblots showing shedded S1 subunits, hIgG Hc, full-length spike, S1, S2 and cleaved S2’ collected from supernatant and cell lysate fractions of HEK293T cells expressing spike WT or Delta VOC stimulated with 12.5 nM CB6 antibody for 16 hours. Data are representative of five repeats. (TIF) [file ppat.1011789.s003.tif]

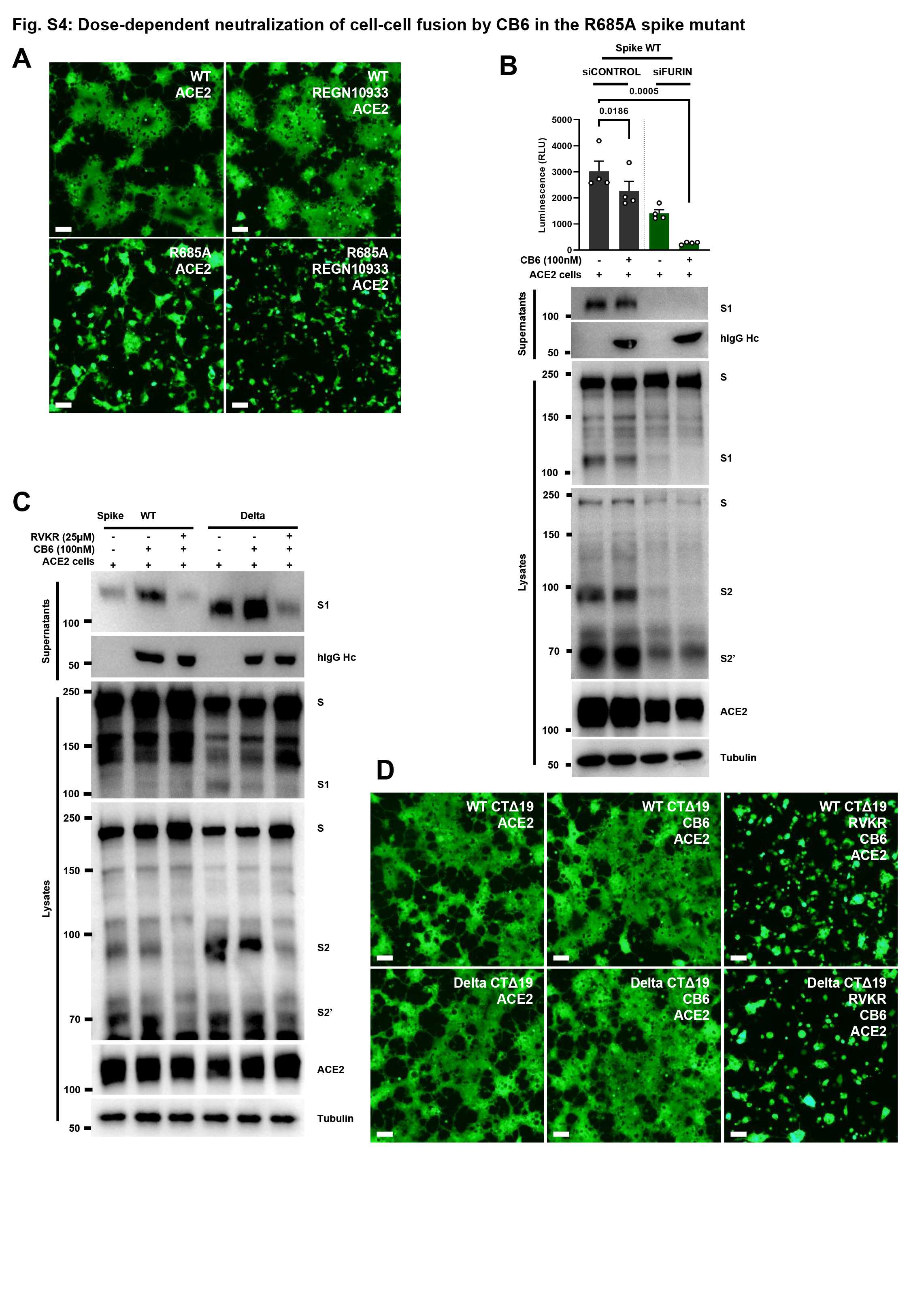

Supplement: S4 Fig — (A) Fluorescent images of REGN10933-mediated neutralization of cell-cell fusion in HEK293T cells co-expressing ZsGreen reporter with WT or R685A spike mutant, preincubated without or with 100 nM REGN10933 for 1 hour, then co-cultured with HEK293T cells expressing ACE2 for 16 hours. Scale bars are indicative of 50 μm and images are representative of two independent experiments; (B) Luciferase activity (RLU) measured from HEK293T cells co-expressing Cre, siControl or siFURIN with WT spike pretreated with 100 nM CB6 for 1 hour, before mixing with Stop-Luc-expressing cells carrying ACE2 for further 6 hours (top); and immunoblots showing shedded S1, hIgG Hc, full-length spike, S2 and cleaved S2’ collected from stimulated cell supernatants and lysates (bottom). Data are representative of four individual repeats, blots are representative of two independent experiments; (C) Immunoblots showing shedded S1 subunit and hIgG IgG Hc, full-length spike, S1, S2 and cleaved S2’ collected from supernatants and lysates of HEK293T cells co-expressing ZsGreen reporter with WT and Delta spike VOCs, preincubated without or with 100 nM CB6 and 25 μM RVKR for 1 hour, then co-cultured with HEK293T cells expressing ACE2 for 16 hours. Blots are representative of two individual repeats; (D) Zsgreen fluorescent images of CB6-mediated neutralization of cell-cell fusion described in (C). Scale bars are indicative of 50 μm, images are representative of two independent experiments. (TIF) [file ppat.1011789.s004.tif]

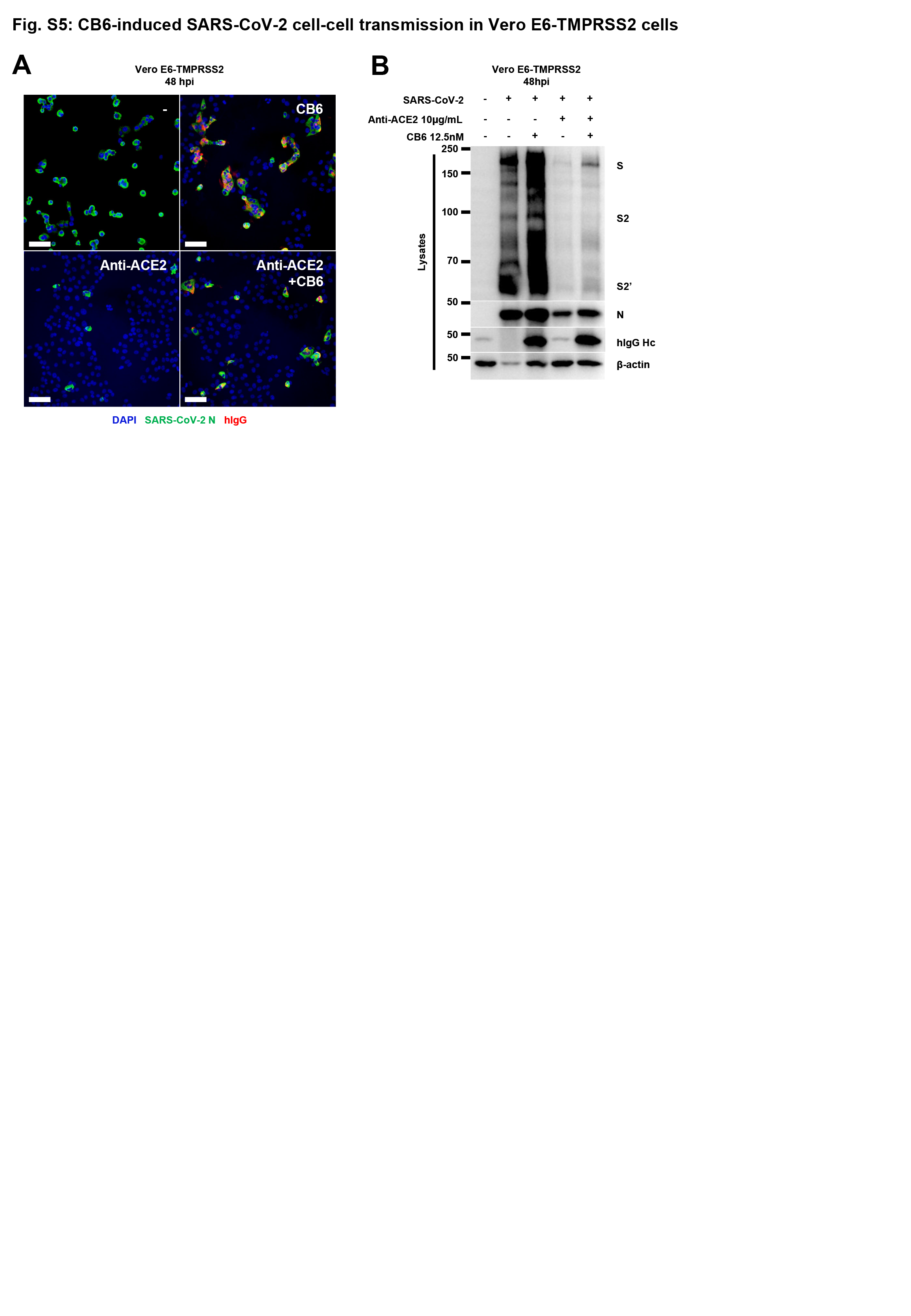

Supplement: S5 Fig — (A) Immunofluorescent images showing morphology of 2 MOI SARS-CoV-2 infected Vero E6-TMPRSS2 cells, without or with 1 μg/mL Anti-ACE2 blocking antibody, treated without or with 12.5 nM CB6 antibody 48 hours post infection (hpi). Anti-SARS-CoV-2 N and Anti-human IgG (H+L chains) were stained with Alexa fluor 488 and 555 respectively. Scale bars are indicative of 50 μm; (B) Immunoblots of SARS-CoV-2 full-length spike, S2, S2’ and N, collected from Vero E6-TMPRSS2 lysates described in (A), blots are representative of two individual repeats. (TIF) [file ppat.1011789.s005.tif]
