# Supplementary material for: Antibody-mediated spike activation promotes cell-cell transmission of SARS-CoV-2
Source: PLoS Pathog. 2023 Nov 10;19(11):e1011789. doi: 10.1371/journal.ppat.1011789 (PMC10664894; doi:10.1371/journal.ppat.1011789)
Supplement: Attachment — Submitted filename: PP_Yu_Review.docx [file ppat.1011789.s006.docx]

In this study, Yu and colleagues investigate how RBM-targeting antibodies, particularly CB6, can activate the spike protein resulting in cell-cell fusion. They demonstrate that binding of class I antibodies can trigger syncytia formation in several cell lines including 293T and A549 expressing low levels of ACE2. They also show that class I antibodies trigger increased S1 shedding that is accompanied by the spike cleavage including at the S2’ site. They show that the binding of class I antibody CB6 to the spike protein causes a conformational change that is likely associated with post-fusion state of the spike. They conclude that the phenotypes of increased syncytia formation, increased shedding, increased cleavage at S2’, and the conformational change are reliant on furin cleavage at the S1/S2 boundary by using a spike with an R685A change or a furin cleavage site deletion, as well as knockdown of furin or by an inhibitor against furin.

While overall the study is well designed and the data is interesting, there are critical weaknesses that must be addressed. First, the role of ACE2 in CB6-mediated cell-cell fusion cannot be completely ruled out, as the cell lines used, including 293T and A549, still express a low level of ACE2. The author should have completely depleted ACE2 in these cells, or use cell lines that are known to express no ACE2. Second, throughout the study, the surface expression of spike is not examined. This is important, given that cell surface expression of spike critically determines cell-cell fusion and is also impacted by S1 shedding. Third, in multiple locations, the author tries to link cell-cell fusion results to cell-cell transmission; however, cell-cell transmission is distinct from cell-cell fusion, and carefully designed cell-cell transmission assay shall be performed to support their conclusions. The use of “virus-to-cell transmission” in the text is also unconventional and confusing. Fourth, while live SARS-CoV-2 virus is used resulting in CPE in Vero E6 cells, no definitive conclusion can be drawn from this experiment, because no clear cell-cell fusion is observed in the presence of CB3, not it is tested in ACE2-deficient cells or using furin-deficient SARS-CoV-2 viruses. The author claim that the CB6 did not prevent the formation of CPE induced by SARS-CoV-2 virus, but the opposite was shown in Fig. 6. Given SARS-CoV-1 does not contain a furin cleavage site, which can serve a perfect control; unfortunately, SARS-CoV-1 is not tested nor mentioned throughout including in the Discussion. The discussion appears open-ended and should be extended to explain the mechanisms of action regarding role of furin, which is normally present in trans-Golgi but not on the plasma membrane, as well as implications for COVID infection and pathogenesis.

Specific comments are below:

1. Throughout the manuscript, no quantifications of western blotting and microscopic imaging are performed. While S1 shedding data is shown, the S cleavage with ratios of both S1/S and S2/S in cell lysates should be examined and shown in parallel.
2. Fig. 2C, no cell lysate data is presented, which is critical. Based on Fig. 2A in 293T cells, CB6 didn’t induce the spike cleavage at S1/S2 site; the author should check and ensure all statements are correct throughout the manuscript.
3. Fig. 6: this is one of the most critical experiments, but unfortunately it was not carefully designed – why was Vero E6 cell chosen? And would how the results from Vero E6 cells reflect that from cells expressing low ACE2? Was the virus produced from Vero E6 sequenced to confirm that there is no deletion in the S1/S2 cleavage and other sites? And how S1 shedding correlated with CPE or cell-cell fusion?
4. A number of statements are not supported by experimental evidence; these include: ACE2-deficient HEK293T cells; role of K417N and Q493R in BA1/BA4 binding to CB6; antibody-mediated spike activation “exclusively” promoting the cell-cell transmission of SARS-CoV-2, etc.
5. There is no detailed explanations or discussion regarding S2’ cleavage, which could be mediated by TMPRSS2 and/or Cathepins.
6. Fig. 3C-D: it’s confusing here that the author did not mention about CB6 but included ACE2 in the legend. Are experiments performed in ACE2 overexpressing cells as done in Fig. 4? The conclusion could be different using ACE2 overexpression. Western blotting of cell lysate should be also shown.
7. Fig. S3: why are WT Δ19 and Delta Δ19, rather than their native spikes used? An explanation should be given.
8. Where is EK1C4 peptide used in this study? It is mentioned in the paper.
9. Some incorrect or overstatements should be modified or corrected:
   a. “Others have also reported that spike-mediated cell-cell fusion is resistant to neutralization antibodies, convalescent and vaccinated sera at doses higher than IC90 concentration of virion particles [31, 32, 47, 49]” – this is not true for cell-cell fusion mediated by spike.
   b. “furin cleavage site strongly promotes cell-cell transmission of the virus” – there is no perfect correlation between them, proper references should be provided.
   c. “it is well-known that cell-cell fusion between lymphocytes driven by the HIV-1 envelop is insensitive to neutralization by antibodies or patient anti-sera” – this is not always true.
   d. “The presence of CB6 antibody had no effect on intracellular viral replication and did not prevent the formation of CPE induced by SARS-CoV-2 virus, yet the antibody facilitated the spike cleavage and cell-cell fusion” – this is an overstatement with no convincing data.
