# Supplementary material for: Antibody-mediated spike activation promotes cell-cell transmission of SARS-CoV-2
Source: PLoS Pathog. 2023 Nov 10;19(11):e1011789. doi: 10.1371/journal.ppat.1011789 (PMC10664894; doi:10.1371/journal.ppat.1011789)
Supplement: Attachment — Submitted filename: Responses letter -0922.doc [file ppat.1011789.s007.doc]

**Response letter for manuscript #PPATHOGENS-D-23-00788:**

**Antibody-mediated spike activation promotes cell-cell transmission of SARS-CoV-2**

**Major comments:**

**Reviewer #1**: 1. Some of the conclusion sentences are inappropriate and make reading through the document quite difficult and quite confusing: 1) Line 175 mentions viral infection, when infection was not assessed there. 2) Line 195 mentions RBD abs are related to “cleavage at two proteolytic sites”, when only S2’ cleavage was assessed at this point. S1/S2 cleavage was assessed later. 3) Line 284 concludes that abs bind to S and causes S to be internalized, and this is the mechanism that prevents cell-cell fusion. However, internalization was not assessed here, nor was the causation of cell-cell fusion tested. 4) Line 351 states that abs can activate R685A and deltaRRAR S proteins only when trypsin was added (Fig 5C). This is incorrect because trypsin had no effect on deltaRRAR profile, and CB6 DID NOT activate cell-cell fusion beyond what was already achieved by trypsin. 5) Line 359 states that S1 shedding is responsible for ab-induced cell-cell fusion. This causation relationship was not tested, so please rephrase.

We appreciate these comments. We have re-phrased the indicated text at Line 175, line 195, line 351 and line 359 accordingly.

For Line 284, we now provide additional confocal microscopy fluorescent images (new Fig. 3F) to show RVKR inhibition of S1/S2 cleavage in WT spike resulted in the colocalization of CB6 with early endosome marker EEA1, and inhibited CB6-induced cell-cell fusion, but not the S2’ cleavage collected from the cell lysates (new Fig. 3E).

2. Throughout the manuscript (lines 193, 215, 220, 234, 236), the authors stated CB6 “induced the spike cleavage of S1”. Since none of the data suggested the abs actually induced more cleavage at S1/S2, but instead causes S1-shedding, please rephrase the sentences for accuracy.

We have made the corrections to these lines. Indeed, CB6 antibody triggered spike activation by inducing S1 shedding instead of S1/S2 cleavage. We have now added cell lysate data to show decreased intracellular S1.

3. Throughout the paper, cell-cell fusion correlated well with S2’ cleavage (as shown with WB), except for Fig 2E, where R685A barely fused cells together but it has just as much S2’ cleavage as WT. Please explain this disconnection.

Although we have shown S2’ cleavage occurs only upon functional receptor or RBM antibody binding in this manuscript, S2’ cleavage event actually determines where membrane fusion could occur. For the R685A spike, we now provide updated confocal images (new Fig. 3B), evidently showing the internalization of CB6 IgG, colocalizing with early endosomal (EEA1) and lysosomal (LAMP1) markers. We also tested whether inhibition of furin activity with RVKR in WT spike-expressing cells. Results showed that RVKR does not affect S2’ cleavage in cell lysates (new Fig. 3E), but leads to CB6 IgG internalization through confocal microscopy (new Fig. 3F).

It is also important to highlight that S2’ cleavage is essentially upstream of the membrane fusion processes, which then requires fusion peptide exposure and refolding of S2 subunit into the 6-helix bundle. Cell-cell fusion and syncytia formation mediated by the WT spike likely promoted all these events to happen rapidly at the plasma membrane. To support this hypothesis, we included a new supplementary figure (new Fig. S2B) wherein EK1C4 was used. This HR1 inhibitor blocks CB6-induced membrane fusion by inhibiting the interaction between HR2 and HR1, without affecting the upstream S1 shedding and S2’ cleavage.

4. Please provide a graphical abstract-style figure at the end of the paper. The detail points of this paper can get very confusing. Also, the authors did not propose a mechanism that explains these complicated phenotypes in hACE2-low vs low cells, and in particle neutralization vs cell-cell fusion promotion. Both of these points can be clarified tremendously by adding a conclusion schematic explaining the findings and the proposed mechanism.

We have now illustrated a comprehensive graphical abstract to distinguish antibody-mediated spike activation in S1/S2 cleaved or cleavage deficient cells.

**Reviewer #2**: 1. Throughout the manuscript, no quantifications of western blotting and microscopic imaging are performed. While S1 shedding data is shown, the S cleavage with ratios of both S1/S and S2/S in cell lysates should be examined and shown in parallel.

We have now added cell lysate immunoblots for all WB figures. Quantification of syncytia formation and co-localization from different field of view and individual repeats are shown.

2. Fig. 2C, no cell lysate data is presented, which is critical. Based on Fig. 2A in 293T cells, CB6 didn’t induce the spike cleavage at S1/S2 site; the author should check and ensure all statements are correct throughout the manuscript.

A549 lysate full-length S and supernatant (cleaved) S1 data is now shown side by side by WB (new Fig. 2D).

3. Fig. 6: this is one of the most critical experiments, but unfortunately it was not carefully designed – why was Vero E6 cell chosen? And would how the results from Vero E6 cells reflect that from cells expressing low ACE2? Was the virus produced from Vero E6 sequenced to confirm that there is no deletion in the S1/S2 cleavage and other sites? And how S1 shedding correlated with CPE or cell-cell fusion?

We appreciate the above comments and have now re-designed the live-virus infection experiments. First, we are aware of the possible deletion of S1/S2 cleavage site in spike when passaged ancestral SARS-CoV-2 virus in Vero E6 cells (in the absence of TMPRSS2). We have verified the original full genome sequencing data provided by the Shanghai CDC (Response Figure 1, below), and confirmed that the no mutation at S1/S2 cleavage site was detected. Viral fresh stocks used for infection experiments were p1 and p2. In fact, presence of S1/S2 cleavage of spike can be also demonstrated by original immunoblots showing cleaved spike S2 collected from cell lysates (Fig. 6D).

Response Figure 1

To differentiate the role of endogenous ACE2 expressed by Vero E6 cells, we now included a virus infection experiments, where a mouse monoclonal anti-ACE2 blocking antibody was used after initial virus entry, to fully prevent ACE2-mediated cell-to-cell and virus-to-cell transmission. We confirmed that CB6 is capable of increasing the number of infected cells in both anti-ACE2 treated WT Vero E6 and Vero E6 TMPRSS2 cells (new Fig. 6G and 6H; Fig. S5A and S5B).

**Reviewer #3**: The manuscript is on several occasions difficult to read and revision for grammar and style issues is needed. It is stated on several occasions that the CB6 antibody induces S protein cleavage at the S1/S2 site. However, this statement is not supported by data. Thus, it is demonstrated that CB6 induces shedding of the S1 subunit and S protein cleavage at the S2’ site but no evidence for CB6 induced cleavage at S1/S2 is reported.

After re-analyzing cell lysate data, we have now provided blots for cell lysates and have made the corrections to these statements. Indeed, CB6 antibody triggered S1 shedding instead of S1/S2 cleavage, evidenced by reduced cleaved S1 detected from lysates.

The authors state on several occasions the WT 293T cells were used as target cells in the cell-cell fusion assay to demonstrate ACE2-independent cell-cell fusion. 293T cells express endogenous ACE2, although some batches at low levels, and are not suitable to demonstrate ACE2 independent cell-cell fusion. For this, experiments with ACE2 KO 293T cells and anti-ACE2 antibodies that block S protein binding to ACE2 are essential.

This is a good point, 293T cells after high passage could start to express ACE2. To address this concern, we performed both CRISPR Knockout and anti-ACE2 blocking antibody experiments to show that the effect of CB6 stimulation was independent of endogenous ACE2 (Fig. S2D-S2H).

It is important to show whether the effects of the antibodies CB6 and REGN10987 in figure 1B were concentration dependent. Furthermore, appropriate controls must be shown: Signals obtained with effector cells alone, target cells alone and effector cells without spike mixed with target cells.
We now show dose-dependence effect of CB6 (new Fig. 2C) and REGN10933 (new Fig. S2A); effect of 12.5nM human IgG isotype control has also been included in these experiments and immunoblots. Our cell-cell fusion assay requires the (Donor) Cre recombinase activity in the Acceptor (target) cells to remove the floxed stop codon. Hence, CB6 derived bioluminescence signal requires both donor and acceptor cells seeded in the same well (Response Figure 2, next page).

Response Figure 2. Luciferase activity derived from Spike and Cre co-expressing cells, co-cultured with control stop-luc cells.

Figure 3C-E: It is essential to compare blockade (and overexpression, respectively) of furin in effector cells and target cells with the expectation being that furin blockade in target cells blocks CB6 induced cell-cell fusion. However, from the results section and the figure legends it is unclear what has been done. Finally, it is essential to not only quantify cell-cell fusion but to also investigate cleavage at the S2’ site under these conditions.

For signal detection, the Cre recombinase and floxed-stop luciferase system hassome limitations. Cell-cell fusion quantified by the co-culture system display values derived only from acceptor (effector) cells, since plasmid encoding the stop-luc has to be transfected separately into these cells. In contrast to split-GFP or hibit/lgbit nano-luc systems, our system eliminates non-specific signals derived from leaky or lysed cells.

Regarding the effect of furin knockdown, we have rephased the text in the original manuscript and figure legend. To avoid confusion, data shown in the original text was generated from the *siControl* in both donor and acceptor cells, versus *siFURIN* in both donor and acceptor cells. Similar changes were done for the furin over-expression experiments.

We have also thoroughly examined the effect of furin knockdown and over-expression in either Donor S and Acceptor cells. Results are shown as below:

Response Figure 3. Differences in luciferase activity and spike cleavage compared in donor and acceptor cells with siControl, siFURIN, pcDNA4 and FURIN over-expressing cells.

As mentioned above, the Cre stop-luc luciferase reporter system was only able to capture signal derived from the acceptor cell. These results suggested that knockdown or overexpression of furin in both donor and acceptor cells is important for antibody-induced cell-cell fusion as reflected by luminescence readings, as well as the spike S1/S2 and S2’ cleavage. However, extent of cell-cell fusion could be higher since our system was unable to quantify cell-cell fusion happening between donor cells expressing S only triggered by CB6 antibody. We have included these limitations in the discussions of the updated version.

Figure 4D: It is well established that R685A markedly reduces S protein-driven cell-cell fusion. Were conditions chosen that allowed for comparable cell-cell fusion driven by WT and R685A S proteins in the absence of antibody? If not, is the inhibition of R685 S protein-driven cell-cell fusion by CB6 simply due to the overall low level of fusion activity?

In consistent to our previous publication and other reports, R685A spike lacking the furin cleavage site displayed reduced syncytia formation with ACE2-expressing cells. The morphology of R685A spike-mediated syncytia between ACE2-expressing cells appeared to be single and clustered cells overtime. Amount of cell-cell fusion quantified by our Cre-luc system displayed reduced signal in compare to the WT spike, but not none. CB6-mediated inhibition of R685A spike was not due to low fusion activity, since inhibition of R685A spike was still dose-dependent on the amount of CB6 used and saturates at 10 nM and above; this is in contrast to the WT spike, where CB6 at 50nM displayed only 10-20% inhibition. Fusion activity of R685A spike can be also restored when trypsin TPCK is supplemented and S1/S2 is cleaved (new Fig. 5C & 5D).

**Minor comments:**

**Reviewer #1**:

1. The word “auto-process” was used to describe S1/S2 cleavage. Since S protein does not cleave itself (aka “auto), I suggest omitting the word “auto”. This appeared a few times.

All ‘auto-process’ have been corrected.

2. HEK293T is not hACE2 KO, but instead has a very low level of hACE2. Please acknowledge this in the manuscript.

We have now added ACE2 knock-out and anti-ACE2 blocking antibody data to validate and confirm that antibody-induced spike cleavage and membrane fusion is independent of the potential ACE2 receptor.

3. The paper uses a collection of antibodies (abs), all used at the same dose. What are the affinity differences of these abs, and can these differences potential explain some of the differential effects documented? Please acknowledge.

We have now added the reported binding affinity in approximate KD of each antibody to the WT SARS-CoV-2 spike RBD. Binding affinities of these antibodies were reported by different groups and maybe difficult to compare in parallel. While binding affinity certainly plays a role in term of the spike activation, RBM epitope specificity may also contribute to their ability in term of driving the spike activation, as demonstrated using the Beta spike N417 and K417 reversion (new Fig. 2E and Fig. S2C).

4. The IFA in Fig 1C is too difficult to independently interpret. Maybe this suggests the ab enhancement of cell-cell fusion is modest in the presence of adequate levels of hACE2. Maybe consider relocating this panel elsewhere.

We now provide immunostaining of spike S2 on antibody-treated A549 cells; syncytia formation has now been quantified.

5. Fig 1E and 2D. What are the mCherry+ cells (look like singular cells) in the absence of ab? Please acknowledge.

Expression of WT spike alone produce residual amount of cell-cell fusion, this effect is likely caused by the instability of the spike S1 and S2 subunit; or the very low of endogenous ACE2 expression; this effect is significantly reduced when Omicron spikes are used.

6. Line 194 mentions Fig 2C to contain lysate data. I do not see lysate data.

Lysate WB has been added (new Fig. 2D).

7. Line 298 is stated incorrectly. CB6 did not inhibit S1-shedding, RVKR did.

This sentence has now been corrected.

8. Line 310 concludes that “trypsin alone triggers minimal S1 shedding”, but “minimal” relative to what? Fig 5B does not have a positive control for S1-shedding, hence this conclusion is inappropriate. Should consider rewording or eliminating this statement.

This sentence has now been rephrased. Original WB display full-length and cleaved S1 in cell lysate, as well as S1 in the supernatants in the absence of antibody stimulation.

9. Line 389 states that this paper shows ab-induced S activation is a “significant biological” feature. This paper does not demonstrate “biological”. Please rephrase.

This sentence has now been corrected.

**Reviewer #2**:

1. A number of statements are not supported by experimental evidence; these include: ACE2-deficient HEK293T cells; role of K417N and Q493R in BA1/BA4 binding to CB6; antibody-mediated spike activation “exclusively” promoting the cell-cell transmission of SARS-CoV-2, etc.

Since we previously mentioned the effect of K417N substitution on CB6 binding to the Beta spike, we now provide an experimental data to support our claim. A reversion substitution of Beta N417 back to ancestral spike K417 was sufficient to restore CB6-mediated S1 shedding and cell-cell fusion (new Fig. 2E and S2C). This experiment emphasizes the specificity of antibody binding onto the RBM is required to drive cell-cell fusion.

2. There is no detailed explanations or discussion regarding S2’ cleavage, which could be mediated by TMPRSS2 and/or Cathepins.

We have added comments on TMPRSS2 and cathepsin on antibody-induced S2’ cleavage. Using the authentic virus infection experiments, we now demonstrated that effect of CB6 on infected cells was enhanced in Vero E6-TMPRSS2 cells (new Fig. 6E, S5A and S5B).

3. Fig. 3C-D: it’s confusing here that the author did not mention about CB6 but included ACE2 in the legend. Are experiments performed in ACE2 overexpressing cells as done in Fig. 4? The conclusion could be different using ACE2 overexpression. Western blotting of cell lysate should be also shown.

Figure legends have been updated in the revised manuscript. Data from Fig 3 were all derived from control cells without ACE2. Lysate WB has been added.

4. Fig. S3: why are WT Δ19 and Delta Δ19, rather than their native spikes used? An explanation should be given.

WT Δ19 and Delta Δ19 are full-length spike constructs with C-terminal truncation of 19 amino acids, these spike constructs do not carry a C-terminus ER retrieval and are mainly used for the packaging of murine leukemia virus pseudotyped particles. We used these constructs to compare between Spike VOCs, since our spike VOCs share similar background, hence stated in the figure and the text.

5. Where is EK1C4 peptide used in this study? It is mentioned in the paper.

We have now added the EK1C4 data in the supplementary figure (new Fig. S2B). The peptide fusion inhibitor potently inhibits antibody-induced spike refolding into the 6-helix bundle downstream of the S2’ cleavage; hence, had no effect on antibody-induced spike cleavage.

6. Some incorrect or overstatements should be modified or corrected:

a. “Others have also reported that spike-mediated cell-cell fusion is resistant to neutralization antibodies, convalescent and vaccinated sera at doses higher than IC90 concentration of virion particles [31, 32, 47, 49]” – this is not true for cell-cell fusion mediated by spike.

We have now changed this statement.

b. “furin cleavage site strongly promotes cell-cell transmission of the virus” – there is no perfect correlation between them, proper references should be provided.

This statement has been removed.

c. “it is well-known that cell-cell fusion between lymphocytes driven by the HIV-1 envelop is insensitive to neutralization by antibodies or patient anti-sera” – this is not always true.

We have now changed this statement, line 544-547.

d. “The presence of CB6 antibody had no effect on intracellular viral replication and did not prevent the formation of CPE induced by SARS-CoV-2 virus, yet the antibody facilitated the spike cleavage and cell-cell fusion” – this is an overstatement with no convincing data.

These sentences have now been removed.

**Reviewer #3**:

1. “The latest human codon optimized Omicron was purchased by Genescripts” Please specify.

Omicron BA.1 spike was purchased from Genescripts and subcloned into pVAX1 plasmids with 19 amino acid deletion at C-terminus to match the expression of other spike VOCs.

2. Please provide source and references for the plasmids used to quantify cell-cell fusion.

We have now added the source of our Cre/stop fusion system plasmids in the methods and added the references for HCV-mediated cell-cell fusion quantification, Reference 66: Si Y, Liu S, Liu X, Jacobs JL, Cheng M, Niu Y, et al. A human claudin-1-derived peptide inhibits hepatitis C virus entry. Hepatology. 2012;56(2):507-15. Epub 20120611. doi: 10.1002/hep.25685. PubMed PMID: 22378192; PubMed Central PMCID: PMCPMC3406249.
